# Ultradian and circadian rhythms of phototaxis in chlamydomonas reinhardii

**DOI:** 10.1016/j.bbrep.2025.102360

**Published:** 2025-11-15

**Authors:** Helen A. Jenkins, Alun L. Lloyd, Jackelyn M. Kembro, David Lloyd

**Affiliations:** a Department of Microbiology, University College, Newport Road, Cardiff, CF24 0TB, UK; b Biomathematics Graduate Program and Department of Mathematics, North Carolina State University, Raleigh, NC, 27696, USA; c Instituto de Investigaciones Biológicas y Tecnológicas (IIByT, CONICET-UNC), Consejo Nacional de Investigaciones Cientificas y Técnicas (CONICET), Córdoba, Córdoba, Argentina; d Instituto de Ciencia y Technologia de los Alimentos (ICTA), Facultad de la Ciencias Exactas, Fisicas y Naturales, Universidad Nacional de Córdoba (UNC), Córdoba, Córdoba, Argentina; e School of Biosciences, Sir Martin Evans Building, Cardiff University, Cathays Park, Cardiff, Wales, CF10 3AX, UK

**Keywords:** Ultradian, Circadian, Rhythms, Oscillations, Phototaxis, Chlamydomonas, GaMoSec wavelet analyses

## Abstract

We investigated phototactic and circadian rhythmicity in Chlamydomonas reinhardii under photoautotrophic (HSM) and photoorganotrophic (HSA) conditions to explore the influence of metabolic context on rhythmic behavior. Cultures were grown under controlled conditions (LD 12:12, 28 °C). Phototaxis was monitored using an automated multi-channel system that continuously recorded light transmission changes associated with photoaccumulation. Cell density and chlorophyll *a* were also quantified. Organisms incubated in continuous dim light showed a robust free-running circadian rhythm (∼24 h) with phase-dependent responses to prior light–dark entrainment, with the maximum rate of phototaxis occurring during the subjective day. The phototaxis rhythm could also be initiated by a decrease in light intensity, and showed temperature compensation for organisms in photoorganotrophic and phototoautotrophic growth media respectively over a temperature range 17°- 28 °C. Time series of phototactic activity were analysed using the GaMoSEC framework—a multiscale wavelet-based approach for identifying periodicities and quantifying rhythmic phase and power. The rhythm persisted longer under photoautotrophic conditions and was temperature-compensated (Q10 of 1.01 for HSM- and 1.07 for HSA-grown organisms). Wavelet analyses also revealed faster oscillations (60–80 min) superimposed on the circadian component, particularly under photoorganotrophic growth. These findings demonstrate that phototactic rhythmicity in *C. reinhardii* is influenced by metabolic conditions and light history and that GaMoSEC analysis effectively captures multiscale temporal organization in biological time series.

## Highlights

• A single drop in light intensity initiated free-running circadian rhythms of phototaxis.
• Temperature compensation was observed for circadian rhythms between 17° and 28 °C.
• Ultradian rhythms of 60–80 min were most evident in cells growing photoorganotrophically.
• Phototactic rhythmicity is influenced by metabolic conditions and light history.

## Abbreviations

CT: circadian time
Cwt: Continuous wavelet transform
‘GaMoSEC’ analysis: a 5-step resolution procedure for resolution of complex rhythmic performance
HSM photoautotrophic: ‘high salts medium: (3/10 strength)’
HSA photoorganotrophic: 15 mM Na acetate-trihydrate containing medium (Sueoka et al., 1967)
Subjective day: subjective night LD: 12: 12: light, dark; 12 h, 12 h ☐, ■
Q_10_: Temperature coefficient
T: Period of oscillation
ZT: Zeitgeber time

## 1 Introduction

Unicellular protozoa and algae have been used extensively to provide systems for studies of the biological clock [1,2]. Of those most commonly employed, each shows different advantages and disadvantages as an experimental system. The large cell size of the green algal species *Acetabularia mediterranea* enables measurements on single cells [3,4]. *Lingulodinium polyedra*, a marine motile photosynthetic dinoflagellate, provides easily monitored rhythms of bioluminescence [[5], [6], [7]]). *Euglena gracilis*, a freshwater microscopic alga, provides an excellent system for investigation of the circadian control of the cell division cycle [[8], [9], [10]]. This latter organism also shows temperature–compensated circadian rhythmicity of its phototactic response [[11], [12], [13]], but preparation of synchronous cultures of either *E. gracilis* [14] or of *L. polyedra* using size selection are not readily achieved (Lloyd, D., unpublished). *Chlamydomonas reinhardii*, a photosynthetic flagellated single-cell green alga, has many advantages. It shows an ultradian rhythm in accumulated chlorophyll *a* in addition to the circadian rhythm observed in cultures growing in light and dark cycles [15]. Synchronous cell division, usually induced by repetitive cultures light and dark cycles, can also be monitored after selection of small cells by a gentle centrifugation procedure [16]. This organism also shows circadian rhythms in its phototactic response [17,18], which persist in the complete absence of external time cues [19]. Furthermore, the isolation of mutants of *C. reinhardii* that show altered circadian clock period lengths make this organism suitable for genetic analyses [[20], [21], [22]].

Whereas circadian phenomena have dominated the literature on biological rhythms for the past 70 years, there is currently an upsurge in the understanding of the ubiquity and importance of ultradian rhythms [[23], [24], [25], [26], [27]]. Detecting and characterizing biological rhythms requires specialized analytical approaches, as biological time series are often noisy and non-stationary (i.e., their mean values and variance change over time). Wavelet-based methods are particularly well suited for this purpose, allowing the identification of transient or evolving rhythmic components across multiple time scales. Recent advances in this area have built upon robust frameworks such as those introduced by Kurz et al., 2017 [28], applied to avian behaviour by Guzman et al., 2017 [29] and further formalized into the 5-step ‘GaMoSEC analysis’ by Flesia et al., 2022, [30]. Kembro et al., 2023 [31] have clearly revealed that matching rhythms, as characterized across evolutionarily distant species during evolution, indicate coherent dynamic features, i.e., for a spontaneously synchronized fermentation yeast (*Saccharomyces cerevisiae*), mice, rats, and Japanese Quail (*Coturnix japonica*). Furthermore, Kembro et al., 2023, 2024 [31,32] have demonstrated that GaMoSEC analysis can effectively detect changes in rhythmicity over time, including phase shifts and the consolidation of a circadian rhythm.

Holography using a lens-free microscopy technique provides a novel method for detection of ultradian rhythms independent of circadian rhythms, as spectacularly demonstrated for cultured mammalian cell lines [33]. This unique application tracks thousands of cells in real time for single-cell dynamics thereby avoiding the need for synchronization of populations, and artefacts derived from labelling. Here we further confirm that novel methods for resolution of complex waveforms in living organisms can help resolve the functional aspects of *C. reinhardii* rhythms.

## 2 Materials and methods

### 2.1 Organisms and growth media

*C. reinhardii* (11/32b. G. M. Smith No. 137c, mating type minus) was obtained from the Culture Collection of Algae and Protozoa, Ferry House, Ambleside, Cumbria LA22 OLP, U.K. Cultures were grown either on a photoautotrophic high salts medium (3/10) strength (‘HSM’) or on a photoorganotrophic 15 mM Na acetate-trihydrate containing medium (‘HSA’) [34].

### 2.2 Culture conditions

*C. reinhardii* was grown in 500 ml conical flasks containing 100 ml medium, and incubated at 28 °C in an LH Incubator (Mark X, LH Engineering Company Ltd., Stoke Poges, England) with orbital shaking at 100 cycles/min under constant light of 13000 lux (approximately 194 μmol photons/m^2^/s) These photoautotrophically-growing organisms were subcultured every 4–5 days by inoculation of 10 ml of culture into fresh medium. Cells growing in the presence of acetate were sub-cultured every 1–2 days. Stationary phase populations were 4–5 and 6.6 x 106 organisms/ml under photoautotrophic and photoorganotrophic conditions, respectively, as determined by haemocytometer counts as were synchrony indices: for details see Jenkins et al. [14,15]. These measurements indicate that acetate inclusion in the medium considerably stimulates the population growth yield and biomass of this photosynthetic organism, a major factor in most commercial enterprises.

### 2.3 Measurement of phototaxis

**Fig. 1:**
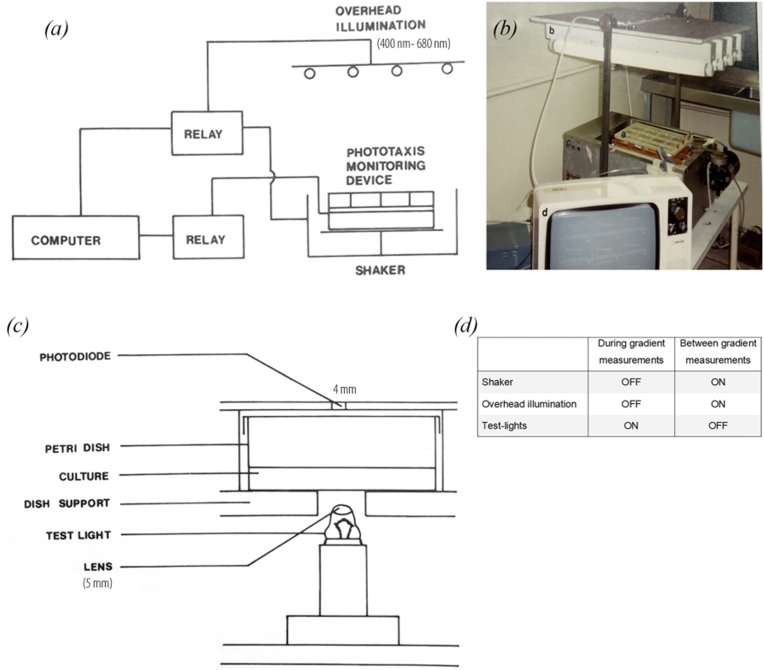
Representation of the experimental setup. (a) The apparatus used for measuring phototaxis: the complete system. (b) photograph of the setup: The phototaxis 4 x 3 array of Petri dishes containing the samples of Chlamydomonas reinhardii in position on the reciprocal shaker under the overhead strip lights illuminating the test samples, (c)A single channel, and (d) table indicating the state of the apparatus used for the sequence of operation during and between gradient measurements.

The equipment used (Fig. 1) represents an elaboration of that originally devised by Bruce and Pittendrigh (1956) [11], and updated by Dr. Takao. Kondo (pers. comm.). Samples of culture (5 ml) were aseptically transferred to 12 sterile single-vented plastic petri dishes (diam. 5 cm; depth, 2 cm). These were placed on a specially–constructed tray over 12 test lights (lens-end bulbs; 2.2V, 0.25 A) at a distance of 0.9 cm. This 4x3 array of identical Tungsten filament in glass bulbs were used as sources of identical intensities emitting white light i.e. blackbody radiation, transmitted through silica glass (300nm-2.3 μm), spectrally all colours blue to red (400 nm-IR, 680 nm) in a 3 mm focused to a 1 mm point source at the center of the 4 or 5 mm thick suspension of organisms (depending on the initially controlled set of experiments but uniformly in each of the 5 × 2 cm Petri dishes.

Intensities of transmitted light beams were measured using photodiodes positioned centrally 2.3 cm above the surfaces of dishes (Fig. 1c). The tray was held in a reciprocal shaker under four 40W cool-white fluorescent strip lights in a temperature-controlled dark room. Between measurements the cultures were shaken and illuminated from above, and the test lights were off.

At predetermined time intervals this illumination and shaking were turned off, and the test lights energized (Fig. 1d). Photoaccumulation (due to positive phototaxis into the test beams) produced decreasing light transmission and gave decreased detector outputs. After a preset time (up to 1 min) for equilibration after cessation of shaking, time course of light detection with each of the 12 channels was recorded, displayed and the gradients calculated and stored on disc. Thus phototactic rates were automatically monitored over extended times: the BBC microcomputer (Acorn Computers, Cambridge, England) employed was fitted with a specially constructed relay circuit for control of illumination and shake, using the cassette port. Data collection and operation of test lights were via analogue port. Programmed variables were: time between measurements, delay time, time over which gradient is measured.

Data were plotted on an XY plotter (Watanabe WX 4636, Environmental Equipment (Northern) Ltd., Nantwich, Cheshire, England). Phototaxis traces were obtained for at least 5 days, with measurements recorded every 30 min. Time series for analysis of the GaMoSEC was obtained from these original images using the function: digitize2.mat in Matlab. Due to image resolution and interpolation between data points in the original digitized plots, (i.e. estimation of the approximate temporal resolution of a pixel to estimate the finer resolution image from the 30 min sampling intervals of the experiments). The resulting time series presents a 10min resolution.

### 2.4 Cell counts

Counting employed a Fuchs-Rosenthal haemocytometer slide. If necessary, cells were diluted to roughly 0.3 × 10^6^ organisms/ml using growth medium and fixed with Lugol's solution at a final concentration of 2 %. If any unhatched daughter cells were retained within the parental wall, these were counted as a single cell. At least 300 cells were counted, with each count undertaken in duplicate. For time series, cell counts were performed at 4 h intervals over a 7-day period.

### 2.5 Extraction and measurement of chlorophyll a

Chlorophyll *a* was extracted with acetone according to the method of Gibbs (1962). The specific absorption coefficient of chlorophyll a under the conditions used was 82.0 L/g/cm (,1941). Determinations were performed in duplicate. The chlorophyll *a* concentration was determined using the formula:

$$\left[\text{chlorophyll}\;a\right]\left(\mu g/l\right)=\left(V_{e}/V_{s}\right)-1/1\;\left[A-A_{a}\right]2.43\;f$$

Where V_e_ is the total volume of the acetone extract (ml); V_s_ is the total volume of the sample (l); 1 is the light path (cm); A and A_a_ are the absorbances at 663 nm before and after acidification, respectively; f, is the factor equivalent to the reciprocal of the specific absorption coefficient (a) multiplied by 10^3^. For chlorophyll *a* in aqueous acetone (80 % (w/v) at a wavelength of 663 nm and a at 82.0 l/g/cm as calculated by both MacKinney (1941) and Zscheile et al. (1942) [35,36].

For time series, chlorophyll measurements were performed at 4 h intervals over a 7-day period.

### 2.6 Fluorescence spectra

Fluorescence spectra of whole cells and acetone extracts were obtained using a fluorescence spectrometer with 150W Xenon arc source and F/3.4 grating monochromator (Applied Photophysics Ltd., London, England). Spectra were recorded at room temperature using a quartz cuvette of 1 cm pathlength.

### 2.7 Measurement of *in vivo* chlorophyll fluorescence

An FM200 filter fluorimeter fitted with a cylindrical silica flow cell (diameter, 3 mm. Baird-Atomic Ltd., Braintree, England) was used. The light source was a fluorescent lamp (4W, length 15 cm) with spectral range 400–500 nm. The excitation wavelength (470 nm) was produced by a double cut-off band blue filter (OB10). Emission was measured at 680 nm using a yellow emission filter (OG1) and a red 645 nm cut-off filter (Baird-Atomic Ltd.)

### 2.8 Data analysis

To detect and characterize oscillations, we applied the first four steps of the GaMoSEC method, as previously described by Flesia et al., 2022 [30], and Kembro et al., 2023 and 2024 [31,32]. In this framework, each time series is analysed sequentially using different wavelet-based transformations (see below for details).

The central idea of wavelet analysis is to compare a predefined mathematical function (the wavelet) with the time series at different time points and scales. This produces a set of coefficients that can reveal not only the presence of a specific pattern but also when in time those patterns occur. When used for detection of periodicities, unlike traditional methods that only capture average frequencies, wavelets such as the complex Morlet continuous wavelet transforms (cwt), allow us to track when rhythmic features emerge, change, or disappear over time. Hence, in plots, the x-axis represents time, the y-axis corresponds to scale (e.g., the period of a potential rhythm), and the coefficient values are shown using a colour scale.

Different types of wavelets highlight different aspects of the signal, so by combining them, GaMoSEC provides a complementary and more complete picture of the underlying dynamics. The method sequentially analyses each time series using the following wavelet transformations:

1 **Gaussian continuous wavelet transform (cwt):** This step is used for visual inspection of fluctuations and irregularities. This transform highlights sudden changes or singularities of a given time scale (e.g., spike- or step-like changes), revealing variability across different temporal scales [32]. In the resulting plots, the y-axis corresponds to scale: smaller scales capture finer details, while larger scales reflect broader, slower dynamics.
2 **Complex Morlet cwt:** This step is applied to detect periodic oscillations. The Morlet wavelet is periodic in nature and generates complex-valued coefficients, which can be decomposed into real, imaginary, modulus, and phase components. The resulting plots, called scalograms, display time on the x-axis, the period of a potential rhythm on the y-axis, and coefficient values on the color scale, which represent the strength of the rhythm at each time and scale. These outputs provide direct evidence of oscillatory behavior and allow detailed characterization of the rhythm. In this study, the real part of the coefficients was used to estimate rhythm phase, as it provides a clear and continuous representation of the rhythm's peaks and valleys [[29], [30], [31], [32]].
3 **Synchrosqueezed wavelet transform:** This step provides highly localized frequency information and is particularly valuable for precise quantification of rhythm period and strength. The method first applies a continuous wavelet transform and then a synchrosqueezing step, which sharpens the frequency representation by concentrating energy around the true oscillatory components. The resulting plots display time on the x-axis, the period of a potential rhythm on the y-axis, and coefficient values on the color scale, which reflect the rhythm's strength at each time and scale. In this study, the modulus of the coefficients is shown, hence periodicity appears as a horizontal band at the corresponding scale. Rhythm power is quantified as the squared modulus of the synchrosqueezed coefficients near the relevant time scale [32].
4 **Empirical Wavelet Decomposition (EWD):** This approach combines wavelet analysis in the Fourier domain with frequency segmentation to extract modal components. EWD can detect rhythms and changes in periodicity, providing an independent confirmation of rhythmic behaviour. The MATLAB toolbox used for this step was developed by J. Gilles [37,38].

The fifth step of GaMoSEC, **Wavelet Coherence**, was not applied here because only a single time series per condition was analysed.

All analyses were performed in MATLAB using custom scripts, which are publicly available on Figshare (2024) [39].

## 3 Results

**Fig. 2:**
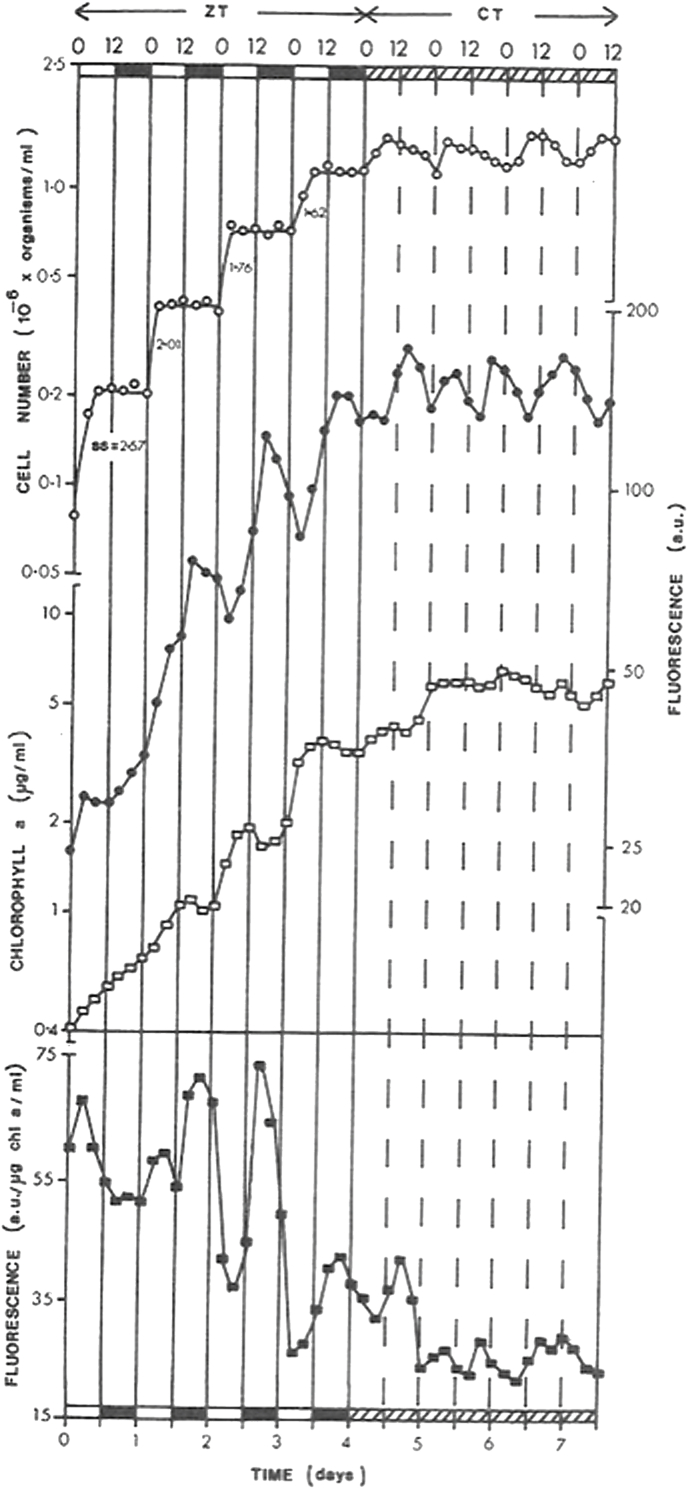
Change in cell number during photoautotrophic growth of Chlamydomonas reinhardii and on subsequent exposure to continuous dim light. Dynamics of (○) cell number; (●) fluorescence spectra of whole cells; (□) extracted chlorophyll a expressed in micrograms/ml and obtained by spectrophotometric determination, and (■) in vivo fluorescence (a.u./micrograms chlorophyll *a*/ml). ZT, Zeitgeber time; CT, circadian time; ss, factorial increase in cell number; light period (13,000 lux); dark period; continuous dim light (3,000 lux).

Synchronization of cell number increases occurred under photoautotrophic conditions (Fig. 2). In this experiment organisms were regularly subcultured for two weeks under alternating light and dark cycles (LD: 12,12) (13,000 lux) before measurements were performed. Cell count showed a high degree of synchrony, increasing during the light phase, while maintaining relatively constant values during each dark phase (Fig. 2). This general pattern continued even after subsequent exposure to continuous dim light. Contrarily, chlorophyll *a* and in vivo fluorescence presented days with peak values during the dark phase (Fig. 2), however after the modification of the light regime this pattern was not maintained.

**Fig. 3:**
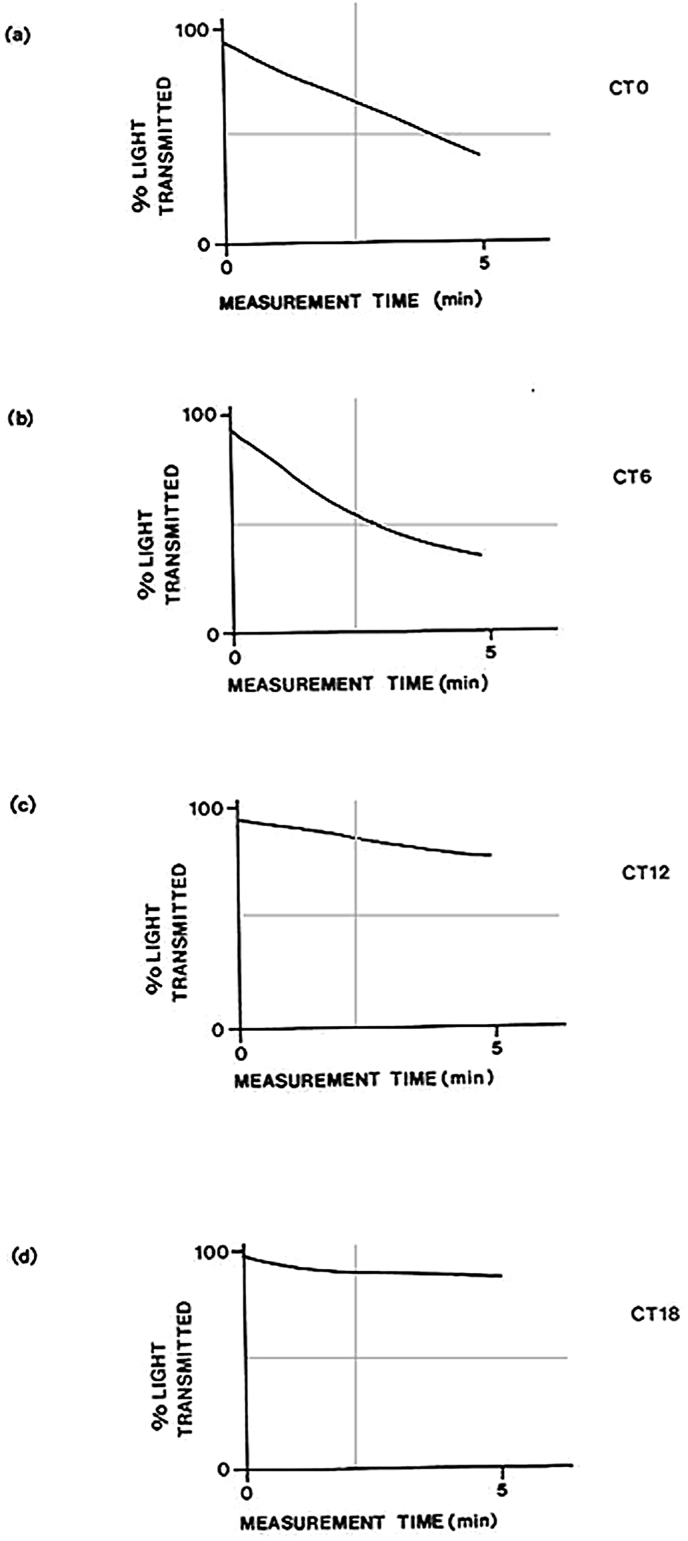
Phototaxis of Chlamydomonas reinhardii measured at different circadian times. Organisms were grown under photoorganotrophic conditions (LD: 12,12; 13,000 lux) using HSM medium before measurements of phototaxis were made under dim continuous illumination (3,000 lux). Photoaccumulation of organisms gradually occluded the test light leading to a decreased photodiode signal. CT0, transition from dark to subjective dawn; CT 6, middle of subjective day; CT12 transition from subjective day to subjective night; CT18 middle of subjective night; temperature, 28 °C throughout.

Fig. 3 shows organisms on HSM under LD: 12, 12 (13,000 lux), that were transferred to the phototaxis measurement apparatus and then maintained under conditions of continuous dim light (3000 lux) and measured every 6 h (CT0, CT6, CT12, and CT18). Photoaccumulation was most rapid at the middle of the subjective day (CT6, Fig. 3b) and lowest at the middle of subjective night (CT18, Fig. 3d).

**Fig. 4:**
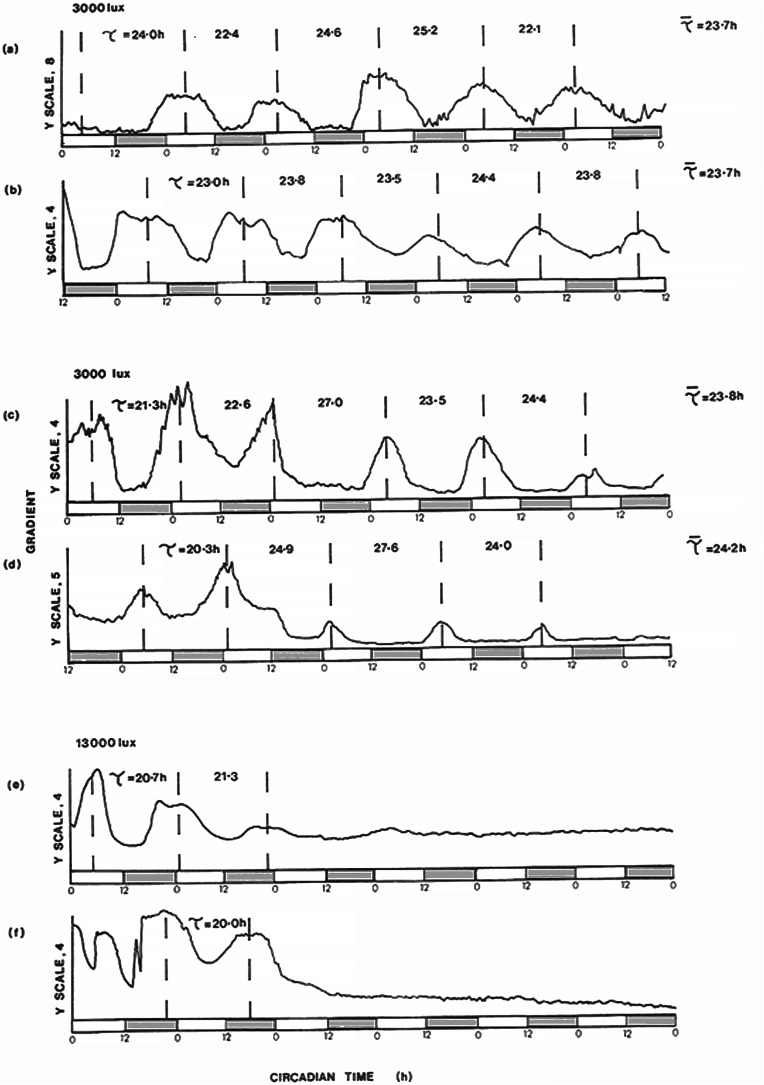
Circadian rhythm of rates of phototaxis in *Chlamydomonas reinhardii* following growth under light-dark cycles (L, D; 12,12; 13,000 lux) synchronized by the LD cycles. Organisms were growing either under photoautotrophic, HSA (a,b,e) or photoorganotrophic conditions, HSA (c, d, f) On transfer to the phototaxis set-up, they were exposed to continuous (a–d) 3000 or 13000 lux from the overhead lights. During measurement of phototaxis (5 min once an hour), these overhead lights would turn off. Note that (a) and (c) were transferred at the beginning of the subjective day, while (b) and (d) at the beginning of subjective night. White/grey bars indicate subjective day L, D: 12, 12: light, dark; 12 h, 12 h ▱ , ▯. Temperature was 28 °C throughout. Waiting time of 30s. Gradient was calculated from the decrease in light transmitted over time. The y-axes refer to appropriately scaled measurement of rates of phototaxis. T values indicate periods of rhythms.

The complete time series of rates of phototaxis over a six-day period is shown in Fig. 4. The free-running circadian rhythms seen in organisms grown on HSM and HSA medium showed phase-dependence on the LD regimes to which they had been exposed during growth (Fig. 4a-d). At 28 °C the free-running period of the circadian rhythm in cells synchronized by previous exposure to LD: 12,12 in both growth media as close to 24 h (in HSM = 23.7 h and in HSA = 24.0 h). The rhythm was more persistent in cells grown under phototrophic conditions (Fig. 4a and b). Also noteworthy on both media the maximum rate of phototaxis occurred during subjective day. Fig. 4e and f shows that the circadian rhythms of phototaxis were lost after 2 days in continuous light (13,000 lux).

**Fig. 5:**
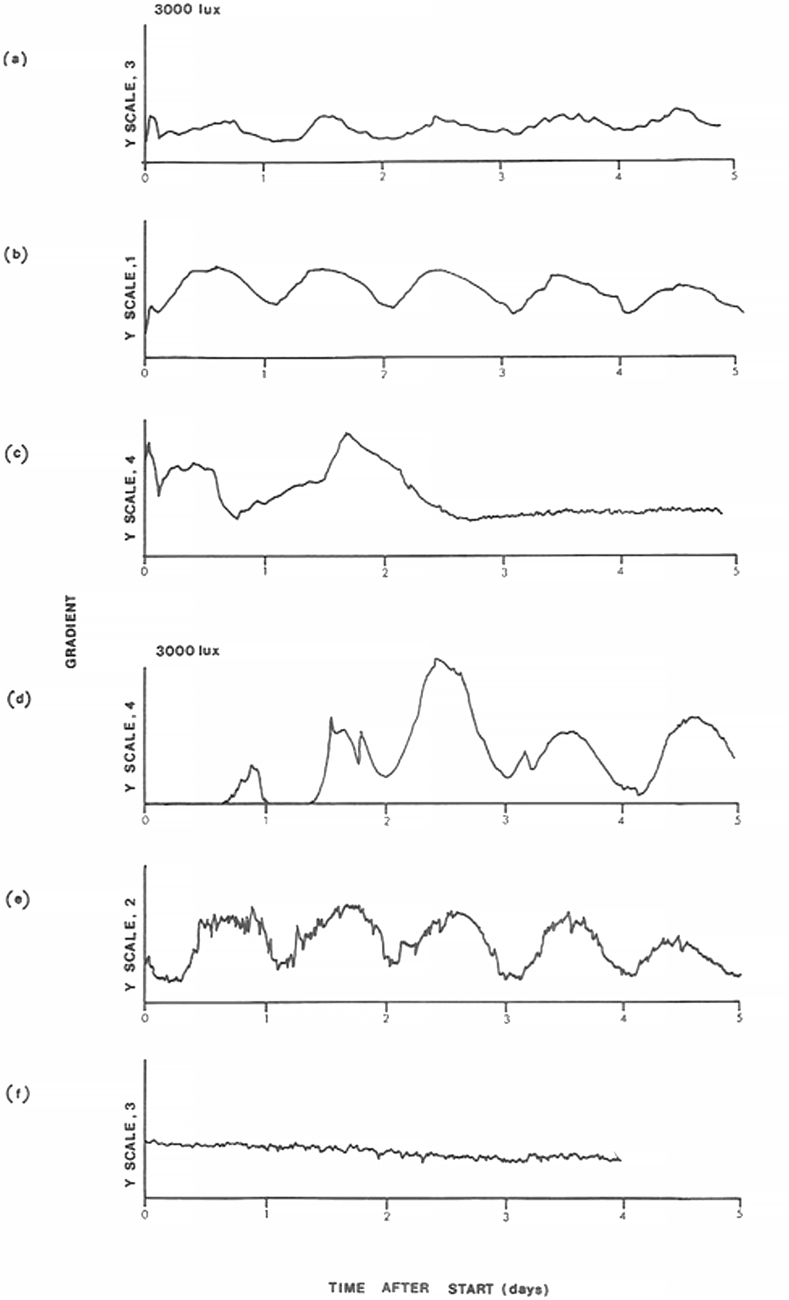
Circadian rhythm of phototaxis in *Chlamydomonas reinhardii* following growth in continuous light (LL, 13,000 lux) under photoautotrophic conditions using HSM medium (a–c), or photoorganotrophic conditions using HSA medium (d–f). Phototaxis measurements were initiated at time 0 at which point, light intensity was decreased to 3,000 lux. Cultures were in early- (a, d), mid- (b, e) exponential or stationary (c, f) phase of growth; temperature, 28 °C throughout.

It was found that exposing C. reinhardii grown under LL, 13,000 lux to a single drop in light intensity to 3000 lux initiated a strong free-running rhythm of phototaxis (Fig. 5). This was so for organisms growing with either HSM (Fig. 5a) or HSA media (Fig. 5d) at early exponential phases of growth. Cells grown to stationary phase on HSA showed no response (Fig. 5f) and those on HSM showed a highly damped rhythm of phototaxis (Fig. 5c).

**Fig. 6:**
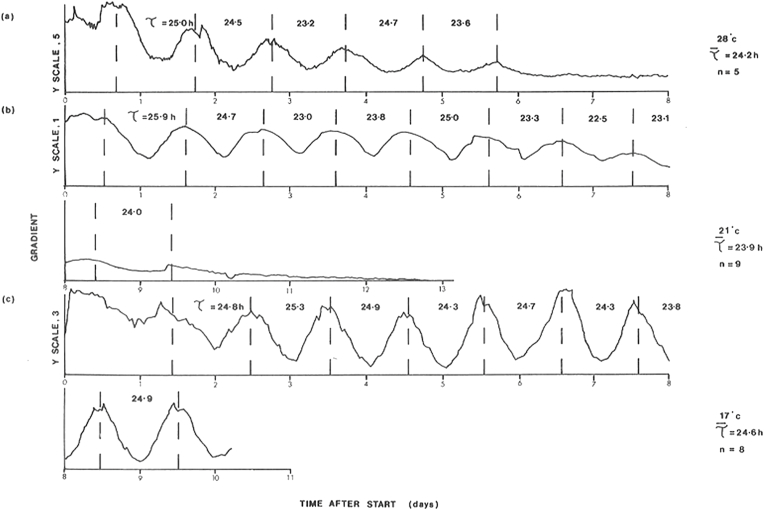
Effect of temperature on the free-running rhythm of phototaxis in Chlamydomonas reinhardii following growth under photoautotrophic conditions using HSM Medium (LL, 13,000 lux; 28 °C). Phototaxis measurement was initiated at time 0 when light intensity was decreased to 3,000 lux and the temperature was either maintained at 28 °C (a), or decreased to 21 °C (b), or 17 °C (c). T values indicate periods of rhythms. Prior to the single drop in light intensity, cultures were grown for at least 10 cell divisions in continuous light of 13,000 lux.

The temperature dependence of the periods of the circadian rhythms of phototaxis initiated by a single decrease in light intensity in mid-exponential cultures of *C. reinhardii* was investigated after growth at 28 °C under LL, 13, 000 lux. Persistence of the rhythms was greater for cells growing with HSM (Fig. 6) than that for HSA (Supplementary Fig. 1), at all temperatures investigated few motile cells remained in HSA after a period of 6 days at 28 °C. On either medium the rhythm persisted longest at 17 °C (Fig. 6, Supplementary Fig. 1). Mean values determined for cells in HSM medium at 28°, 21°, and 17 °C were 24.2, 23.9 and 24.6 h respectively (Fig. 6a–c, respectively), corresponding to a Q_10_ value of 1.01 over that temperature range. For HSA-grown organisms period means were 22.7, 23,5 and 24.2 h at these temperatures (Q_10_ = 1.07).

**Fig. 7:**
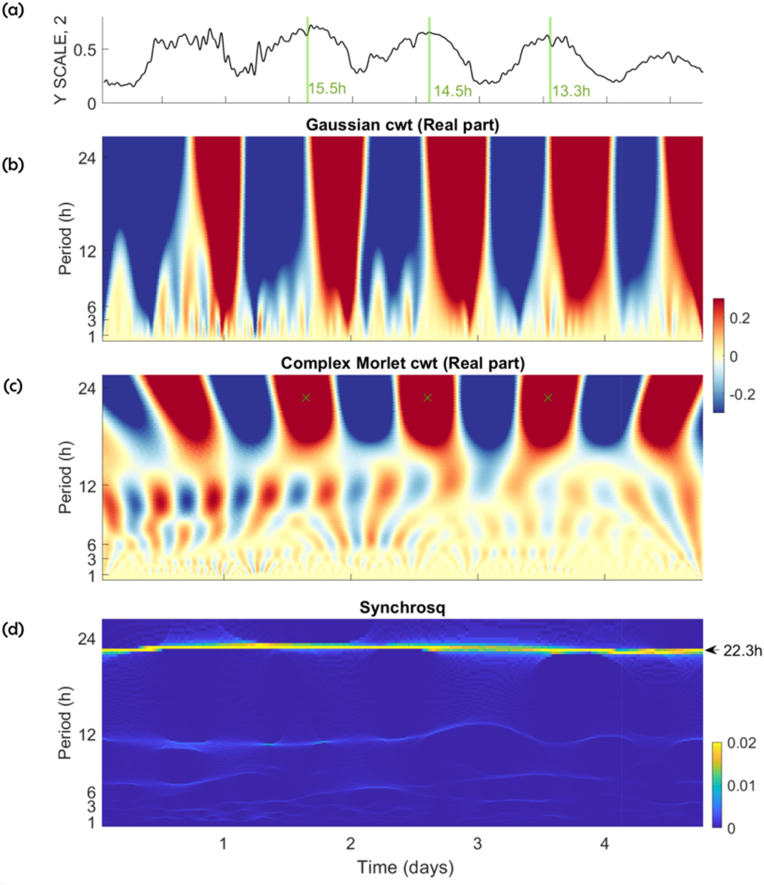
Analysis using GaMoSEC of phototaxis in *Chlamydomonas reinhardii* following growth in continuous light (LL, 13,000 lux) under photoorganotrophicanic conditions using HSA medium (a) Representative time series (same as in Fig. 5e) and the first three steps of GaMoSEC, namely the Gaussian continuous wavelet transform cwt, (b), the real part of the complex Morlet cwt (c) and Synchrosqueezing. A strong daily rhythm is observable at the 24th scale (y axis) with the 3 methods. Peak activity estimated with the Complex Morlet cwt (panel c, ‘‘x’’) is observed daily between 13.3 and 15.5 h showing a strong daily rhythm with peak activity estimated with the between ZT 13.3 and 15.5 h; also indicated in green bars in (a), with a period of 22.8 h as estimated with Synchrosqueezing (yellow horizontal line in panel (d). Fluctuations at shortest time scales are evident as explored in Fig. 7.

Fig. 7 presents an in-depth analysis of the free-running phototaxis time series of the mid-exponential growth culture of *C. reinhardii* grown under photooorganotrophic (HSA medium) in continuous light and exposed to a single drop in light intensity (at Day 0). Visual inspection of the time series reveals not only pronounced oscillatory periodicities of approximately 24 h, but also faster fluctuations occurring at shorter time scales (Fig. 7a, same data as Fig. 5e). To quantitatively detect these rhythmicities and estimate their period and strength, the five-step GaMoSEC approach was applied. The first three steps of this analysis are illustrated in Fig. 7b–d.

The first step consists of the Gaussian cwt (Fig. 7b) which highlights changes in the signal at a given scale. Positive coefficients (shown in dark red) indicate transitions from higher to lower values, whereas negative coefficients (shown in blue) represent the opposite. Accordingly, the dark red vertical bands at the 24 h scale (y-axis) mark transitions from rapid to slower photoaccumulation, while the blue bands correspond to transition from slow to faster photoaccumulation every 24 h. At smaller scales down to approximately 1 h (bottom of panel Fig. 7b,), additional vertical lines become apparent, providing an initial indication of fluctuations at these shorter time scales.

In the second step the real part of the complex Morlet cwt reveals periodic behaviour around the 24 h scale. Positive coefficients (dark red) indicate rhythm peaks, while the negative coefficients (dark blue) mark the “valleys” (Fig. 7c). The alternating red and blue bands thus represent the rhythm's phase, with peak values (in red) occurring between 13.3 and 15.5 each day (Fig. 7c). As with the Gaussian cwt, fluctuations are evident for smaller time scales (see also 8b, as described below).

The third step of GaMoSEC, Synchrosqueezing, provides a precise quantification of the period of the rhythms throughout the test, displayed as a vertical line at the 22.3 h time scales (Fig. 7d). The value of the coefficient reflects the power of the rhythm. In addition to this dominant ∼24 h rhythm, fluctuations at shorter scales are also detectable, though they exhibit lower power and reduced frequency localization (see also Supplementary Figs. 2 and 5).

**Fig. 8:**
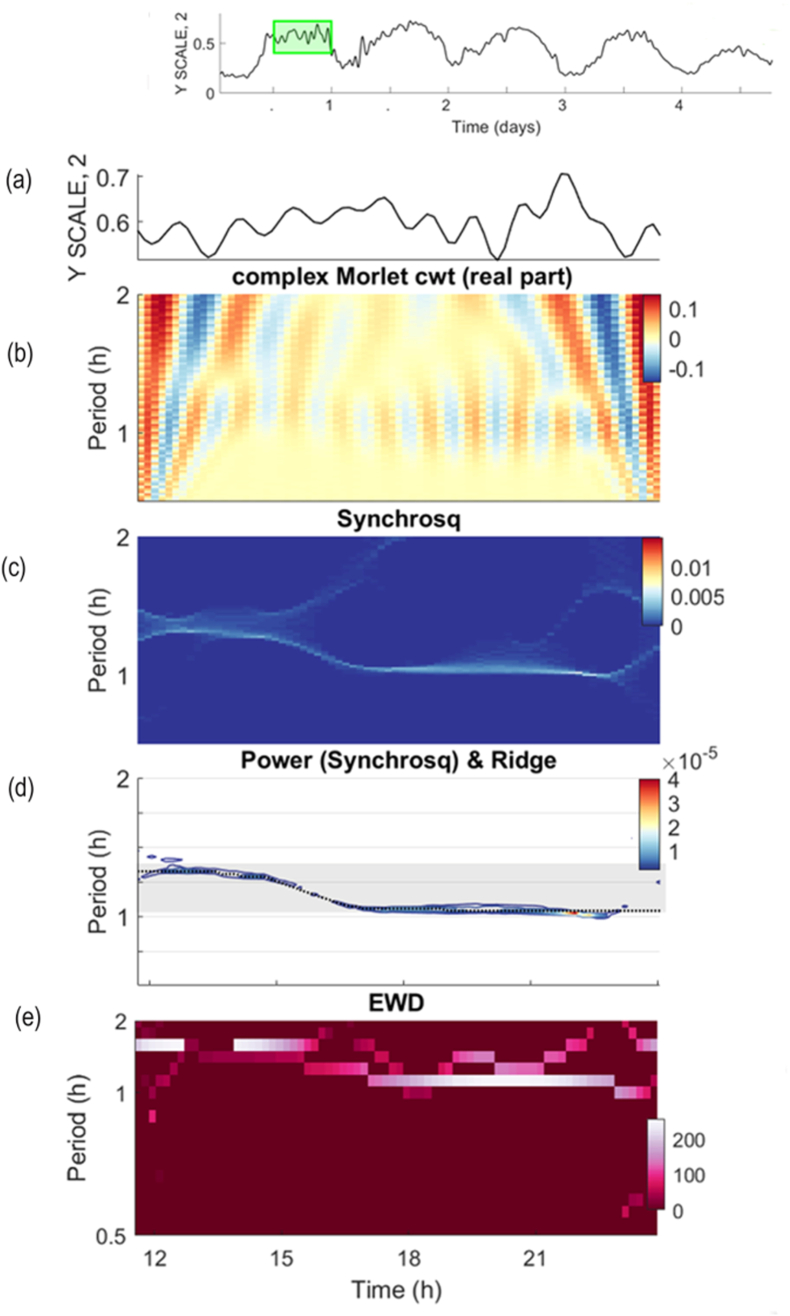
Higher-frequency fluctuations of phototaxis in Chlamydomonas reinhardii. (a) Zoom-in on the representation of the example time series presented in Fig. 5e, and analysed Fig. 7 (inset). Here the Gaussian cwt is not shown (see Supplementaty Fig. 3). (b) The real part of the Morlet shows at the 1 h scale a characteristic pattern of blue (valleys) and red (peaks) in the oscillation. (c) Syncrosqueezing shows that the frequency varies over time. (d) The dotted line shows the ridge marking the estimated period of the fluctuation, with the grey background highlighting the observed range. (e) Empirical wavelet decomposition confirming the fluctuation with a period of close to 1 h.

Fig. 8 provides a closer examination of the higher-frequency fluctuations, offering a magnified view of the analysis shown in Fig. 7. As stated previously, the complex Morlet cwt reveals rhythmic fluctuations in the phototactic time series at scales around 1–2 h (Fig. 7b and c and 8b). The horizontal band in the Synchrosqueezing plot (Fig. 8c and d) provides a precise quantification of the period of the rhythm. In this plot, the ridge connecting the highest values of coefficients estimated for each time point is indicated by a dotted black line (Fig. 8d). Notably, the ridge is not completely straight, slightly decreasing from a 1.3 h period to a 1 h period (grey area in Fig. 8d, equivalent to a range between 60 and 80 min). The power of the rhythm is indicated in the colour scale of the plot (Fig. 8d).

These fast fluctuations (with a period around 1 h) are also evident in the fourth step of GaMoSEC, the empirical wavelet decomposition (Fig. 8e). In this analysis, positive coefficients at a given scale (y-axis), represented as horizontal bands, also indicate periodicity. Note that the decrease in period over time is also observable. Interestingly, these fluctuations are strongest under mid-exponential growth and photoorganotrophic (HSA medium) conditions, with a power around an order of magnitude higher than in the mid-exponential stage with HSM medium or in the stationary phase with either medium (Supplementary Figs. 3–5).

Also, these high frequency fluctuations are not clearly observed in any of the early-and mid-exponential growth phases under photoautotrophic conditions HSM medium (Supplementary Figs. 3 and 5).

## 4 Discussion

The ultradian rhythm with approximately 1 h periodicity as reported by Jenkins et al. (1988, 1989, 1990) has also been observed in *Euglena gracilis* by Adams in 1990) [40], alongside those with shorter and longer period ones (8 min–1.5 h) by Balzer and Hardeland (1993) [41]. Interestingly, control experiments indicated that organisms maintained continuously at a light intensity of 13,000 lux showed no rhythms of phototaxis [14]. Although in our work, the data resolution was insufficient to estimate the exact periods, high-frequency oscillations were ultradian rhythms of 60–80 min were most clearly evident in cells growing photoorganotrophically with Na acetate as determined by the first 4 steps of the GaMoSEC analysis. Future studies including video recordings of cultures as well as higher resolution data is necessary to further confirm the presence of these ultradian rhythms in photoaccumulation, and to further discard other potentially affecting phenomena such as bioconversion or artefacts associated with the repetition of photoaccumulation assays. However, it is important to also recognize that the automated measurements of phototaxis described here provides a convenient means for additional studies of the possible interaction between circadian, cell division and ultradian cycles.

As shown in Fig. 2, cell counts increase during the light phase, while it maintains constant during the dark phase. This dynamics responses to the complex cell cycle of *C. reinhardii* [42,43]. Previous studies have shown that G1/growth occurs during the light phase with replication, mitosis and cell division occurring during the dark phase. The daughter cells are retained within the mother cell wall for a short time before release, with ‘hatching’ occurring at the beginning of the light periods to release up to 4 daughter cells through breakdown of mother cell walls [34]. Interestingly, herein the rhythm of cell division observed under LD conditions persisted under constant dim light conditions (LL, 3000 lux). Growth under photoorganotrophic conditions with HSA (not shown) showed qualitatively similar characteristics with respect to the persistence of this induced rhythm under constant illumination [14]. In order to optimize the sensitivity of measurement of the rate of phototaxis, several different conditions were tested. A waiting period of 30s after the shaker had stopped was found to be adequate to obviate initial disturbance of photoaccumulating organisms. Measurement times of more than 5 min were found to raise the temperature of the cultures and lead to condensation on the Petri dish lids. For studies of circadian rhythms, the interval between measurements was usually 20 or 30 min. It has previously been shown that the photochemical action spectrum for phototaxis has maxima at 503 and 443 nm [44]. That the organism does not respond to red light has been confirmed by using a red filter (cut off at 590 nm) [45]. However, the phototactic responses obtained using a blue filter (⎣max = 475 nm) in the test beam were not enhanced over those with white light. An intensity of 1000 lux gives maximum phototaxis, and photoinhibition begins to occur at somewhat higher intensities [45].

When the period of an oscillation is temperature compensated it potentially can act as a stable intracellular time base for synchronization of concurrent processes. Noteworthy, this property is distinctive since neither cell division cycles nor glycolytic oscillations have been shown to exhibit temperature compensation. Therefore, this type of oscillatory behavior [46,47] can be regarded as an indication of a biological timekeeping function.

By analogy with the daily rhythms which are controlled by the circadian clock, higher-frequency oscillations are often referred to as manifesting ‘an ultradian clock’ [16,40], but more accurately in functional terms as a series of ‘metronomes’, or ‘ultradian timekeepers’ [24,48]. Time resolution of the very many ultradian intracellular processes of cellular growth has thus revealed a system of timed coupled multi-oscillators [49].

The ‘master’ oscillator of the cellular system, in a model system can be considered analogous to a more massive pendulum bob [50] determining the overall frequency of the coupled system [51]. These ultradian oscillations based in the ‘epigenetic’ time domain [52] originate in the complex and extensive system feedback loops that result from temporal changes in the control of gene expression. Specifically, they involve transcription, translation, and perhaps modification of proteins. In regard to respiration, energy-yielding reactions are enslaved by this ‘master oscillator’, hence they do not exhibit their own characteristically rapid dynamics; instead, they also present approximately hourly cycles. “So the cell's ‘slow dance to the music of time’ has the fast mitochondrial activities entrained to an epigenetic ‘piper’ that plays a slower tune” [15]. Thus the dynamics of energy demand is predominate, and the energetic capacity is never limiting. This close connections between energy demand and its supply are provided by the ATP/ADP pool (analogous to the linking to a multiplicity of pendulums). Thus ‘growth’ of organisms, usually still usually considered as being a continuous process ‘under homeostatic controls within limits’ is a widely used term, but is more precisely defined as ‘homeodynamics’ as originally specified by the distinguished physiologist, Eugene Yates, please see Ref. [53]. Neglect of the underlying dynamic complexity of the faster time-scales of the integrated development within cells (from cellular differentiation, and the cell division cycles through transcription, translation, protein modification, metabolism, and even faster molecular interactions of cell-cell signalling and functional change still largely evades understanding.

Many of these basic principles were initially gleaned from studies on the respiration of synchronous cultures of *Schizosaccharomyces pombe* [54] then from two other yeasts and eight protists, including *C. reinhardii* and *E. gracilis*, reviewed in Ref. [52]. Comprehending that the entire biochemical network of the model eukaryote effectively does not operate in a tightly homeostatic manner is fundamentally important to transform the understanding of the mode of coherence of the living cell [53].

Cellular molecular architecture and function is robust yet flexible, and can best be understood [55] in terms of network organization and extending to the timekeeping within the genome [56]. It is Systems Biology that represents the paradigm aiming at a whole-organism-level understanding of biological phenomena, whilst emphasizing interconnections [57], and functional interrelationships rather than the component parts. Thereby, studies of network properties, and how they control and regulate behaviour from the cellular to the whole organism level, constitutes its main focus as it addresses from a novel perspective the major still unsolved biological problem.

As molecular and genetic techniques have been developed for *C. reinhardii* it has become an ideal model organism for the study of the molecular mechanism of circadian timekeeping [58]. A search of the nuclear, chloroplast and mitochondrial genomes of *C. reinhardii* for potential homologs to the clock-related genes of other organisms (including *Synechcoccus elongatus*, *Neurospora crassa*, *Arabidopsis thaliana*, *Drosophila melanogaster*, and *Homo sapiens*) revealed that significant similarities were not found with the genes currently considered to be central components of the cellular clock [58]. In most eukaryotes their circadian machinery relies on a mechanism of well defined interlocked transcription-translation feedback loops as well as environmental exogenous factors such as light and temperature [59]. Although the same underlying feedback principle is maintained the key components of the circadian mechanism are substantially different between organisms. Petersen et al. (2022) in their recent review proposed that the circadian clock of flagellate algae may have partially different properties in comparison to the clock of sessile plants and nonflagellate algae. Thus, it is also possible that it will have its own specific ultradian endogenous controls.

The structural complexity of *Chlamydomonas* chloroplasts and branched mitochondria [60,61] changes dynamically over time [61]. Across the circadian cycle, mitochondrial ATP is generated from acetyl-CoA and stored during the dark phase, while NADPH produced by functional thylakoid membranes is maintained to restart photosynthetic energy production in the light [62]. Acetate strongly influences the balance between the glyoxylate cycle in peroxisomes, the mitochondrial TCA cycle, and chloroplast metabolism [63]. Consequently, cross-talk among these organelles is highly intricate and involves multiple overlapping pathways [[64], [65], [66], [67]]. Moreover, in related species the tricarboxylic acid and glyoxylate cycles have been shown to be also absolutely necessary in the assimilation of acetate, propionate and butyrate [[68], [69], [70]]. Further and deeper investigations into the temporal relationships of metabolic interactions [25,26,55] are necessary for more complete understanding of the organellar organization, coherence, control, and indeed the fuller exploitation of the metabolic flexibilities of the green alga, *Chlamydomonas reinhardii*.

Here we present new evidence of key facets of rhythmic phototaxis in *C. reinhardii*, demonstrating the coexistence of circadian (∼24 h) and ultradian rhythms and their modulation by metabolic state and light history. Our results support the contention that complex interactions may occur in lower eukaryotes between the day-time, photosynthetic energy generation of the chloroplast, and organotrophic mitochondrial activities, most specifically when acetate is provided in the HSA medium.

## 5 Future studies

Algal organisms are potentially treasure houses in an age when climate change and the pressing needs for altered energy generation becomes paramount [71]. Novel methods for the genetically-engineered algae make the commercial production of pharmaceuticals, nutracuticals, and prebiotics routine [72], and most recently, radiotheranostics for cancer treatments [73,74]. All this comes in addition to offering novel paths to disposal of chemical pollution from the environment [44]. Production of H_2_ on a research scale from algal cultures is a longstanding laboratory interest and currently an important aim for many commercial groups [75], and large-scale outdoor syntheses of natural carotenoids and engineered bis-abolene [76] many possibilities are as yet hardly exploited. Moreover given the extensive literature on Chlamydomonas spp. the most apposite and extremely detailed, fascinating and important accounts of the analyses of the structures, functions and physical mechanisms involved in the behaviour of the antennae [77], rhodopsin-containing eye spot receptor [78] and two flagella in this tiny (10-20um) long organism in many publications including [[78], [79], [80]] cyclic AMP regulates + or – phototaxis in Chlamyomonas [81]. Moreover, since phototaxis is intrinsically linked to flagellar length and beating patterns/frequency, future studies incorporating these parameters would provide essential mechanistic insight into the rhythmic phenomena observed in our work.

*C. reinhardii* whilst growing in darkness, still await definitive biochemical analyses by complementary documentation of mechanisms involved in acetate assimilation during rhythmic performance for deeper understanding of circadian and ultradian mechanisms, using the GaMoSEC procedure. The rapid rhythmic reactions in the metabolic regimes (and faster in vivo reactions, e.g., those of mitochondrial electron transport) require specially devised equipment, e.g., the flying-spot fluorimeter [82] for oxidised flavins or reduced nicotinamide nucleotides at 104 to 105 data points per second and display at 1–10 times per second. In an early study on cells (an amoeba and a yeast) spread on solid media illustrated the potential for characterization of ultradian rhythms in the minute time domain [83].

## 6 Dedication

To the late Dr. Alan J. Griffiths, (1940–2004) who initiated this research, for his friendship, his deepbiological insights, and his practical experience of culturing protists.

## CRediT authorship contribution statement

**Helen A. Jenkins:** Conceptualization, Data curation, Formal analysis, Investigation, Methodology, Visualization, Writing – review & editing. **Alun L. Lloyd:** Data curation, Formal analysis, Investigation, Methodology, Resources, Writing – review & editing. **Jackelyn M. Kembro:** Data curation, Formal analysis, Visualization, Writing – review & editing. **David Lloyd:** Conceptualization, Formal analysis, Investigation, Methodology, Supervision, Writing – original draft, Writing – review & editing.

## Declaration of generative AI and AI-assisted technologies in the manuscript preparation process

During the preparation of this manuscript, the author(s) used ChatGPT to enhance the visual quality of Figures 2–6 by improving image resolution, whitening backgrounds, and adjusting contrast. After applying these enhancements, the author(s) verified that no modifications were made to the time-series data or axes. The author(s) take full responsibility for the content of the published article.

## Declaration of competing interest

We have nothing to declare.

## Data Availability

Data will be made available on request.
