# Supplementary material for: Ultradian and circadian rhythms of phototaxis in chlamydomonas reinhardii
Source: Biochem Biophys Rep. 2025 Nov 15;44:102360. doi: 10.1016/j.bbrep.2025.102360 (PMC12664618; doi:10.1016/j.bbrep.2025.102360)
Supplement: Multimedia component 1 [file mmc1.docx]

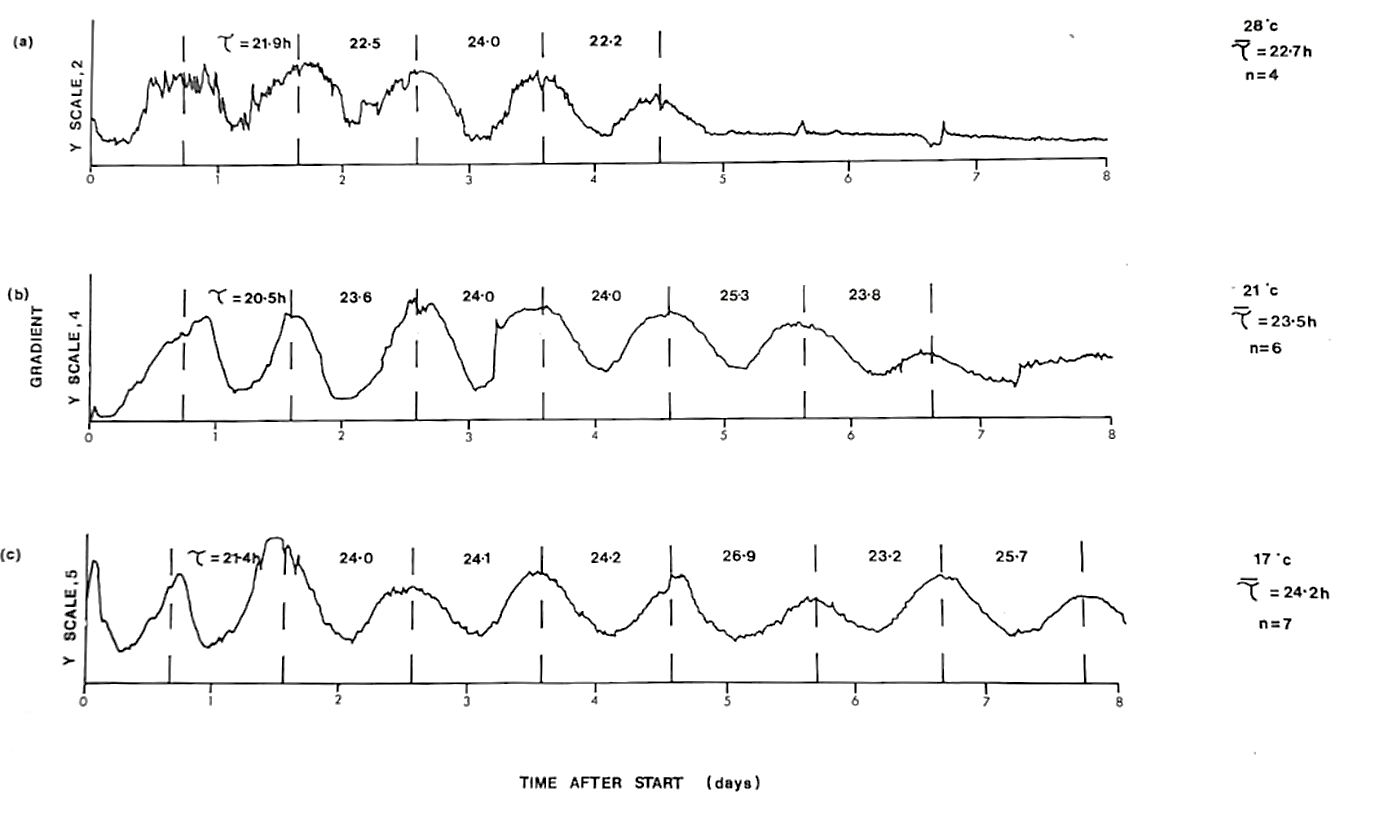


Supplementary Figure 1. Comparison between the effect of temperature on the free-running rhythm of phototaxis in *Chlamydomonas reinhardii* following growth using HAS (d-f) medium (LL, 13,000 lux; 28oC). Phototaxis measurement was initiated at time 0 when light intensity was decreased to 3,000 lux and the temperature was either maintained at 28^o^C (a), or decreased to 21^o^C (b), or 17^o^C (c). T values indicate periods of rhythms.


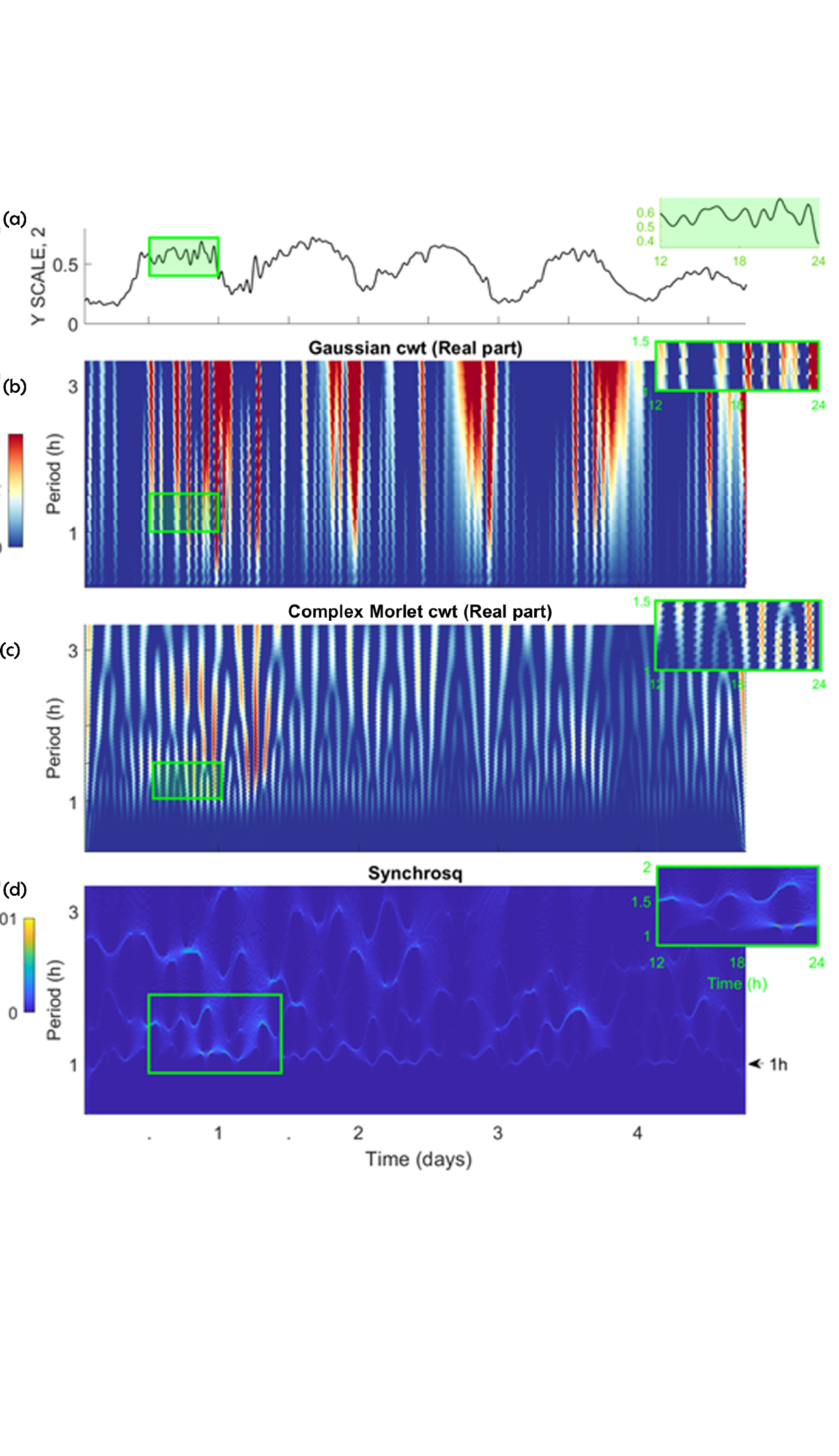


Supplementary Figure 2. A Zoom-in on the representation of the example time series shown in Figure 6 using GaMoSEC of phototaxis in *Chlamydomonas reinhardii* following growth in continuous light (LL, 13,000 lux) under photoorganotrophic conditions using HSA medium (a) Representative time series (same as in Fig. 4e) and the first three steps of GaMoSEC, namely the Gaussian continuous wavelet transform, cwt, (b), the real part of the complex Morlet cwt (c) and Synchrosqueezing. A strong daily rhythm is observable at the 24^th^ scale (y axis) with the 3 methods. Fluctuations in signal are observed down to approximately 1h time scales, although an exact period cannot be obtained (see further analysis of the region selected in green in Figure 7 of main text), original data resolution is 30min.


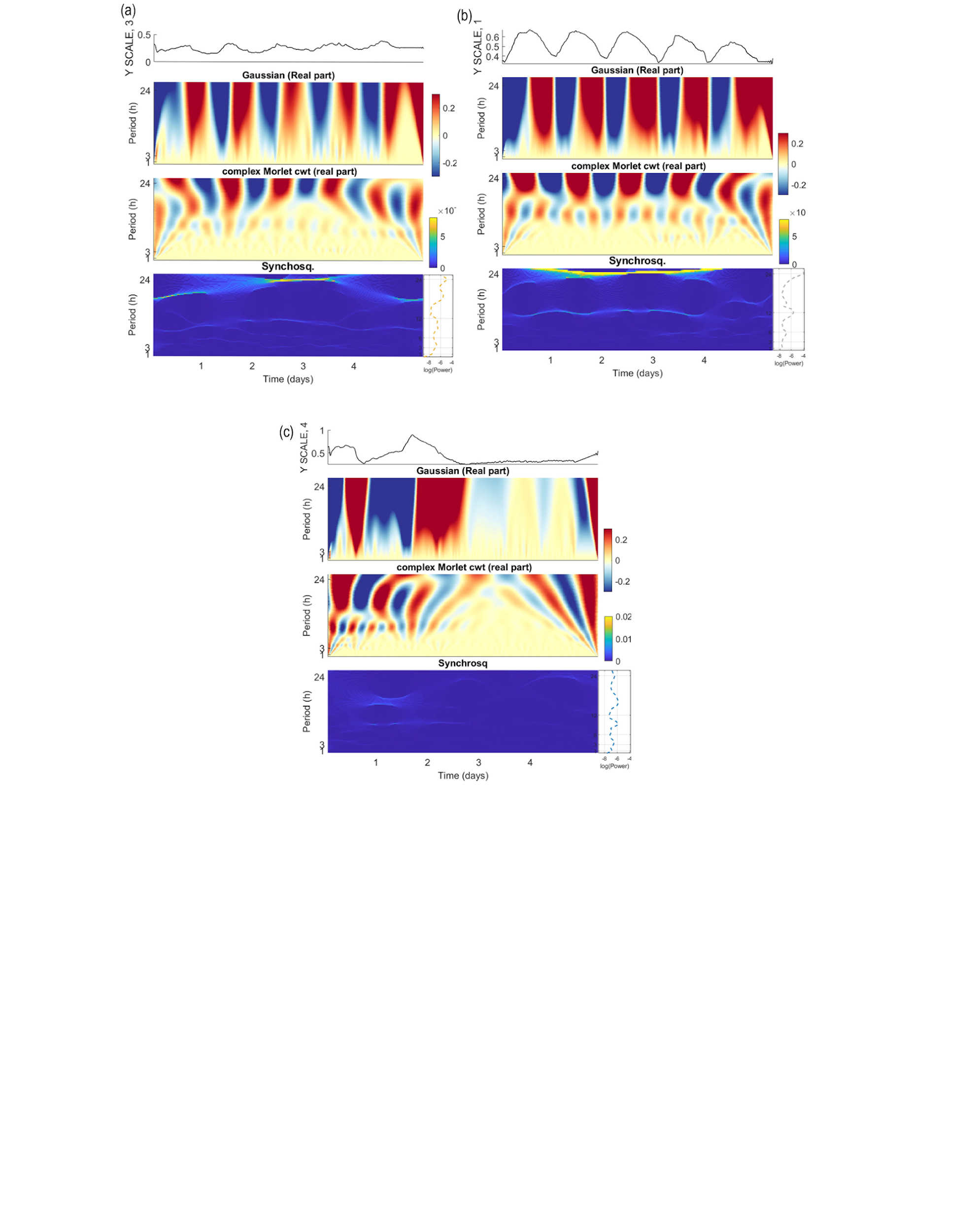


Supplementary Figure 3. The GaMoSEC, method was applied of phototaxis in *Chlamydomas reinhardii* following growth in continuous light (LL, 13,000 lux) under photoautotrophic conditions using HSM medium. Phototaxis measurement was initiated at time 0 at which point light intensity was decreased to 3,000 lux. From left to right, cultures were in early-, mid-, exponential or stationary phase of growth; temperature, 28^o^C throughout. The time series are the same as shown in Fig. 4a-c. For each time series the first three steps are shown, namely the Gaussian continuous wavelet transform, cwt, the real part of the complex Morlet cwt and Synchrosqueezing. In this last analysis, power was estimated for each hour window from the Syncrosqueeze scalogram (see comparison between conditions in Supplementary Figure 4) and shown in the left panel inset. The distinct temporal dynamics of each time series is evident in analysis. Yellow horizontal bands in panels “a” and “b” indicate the ~24h rhythms, which is not observed in panel “c”.


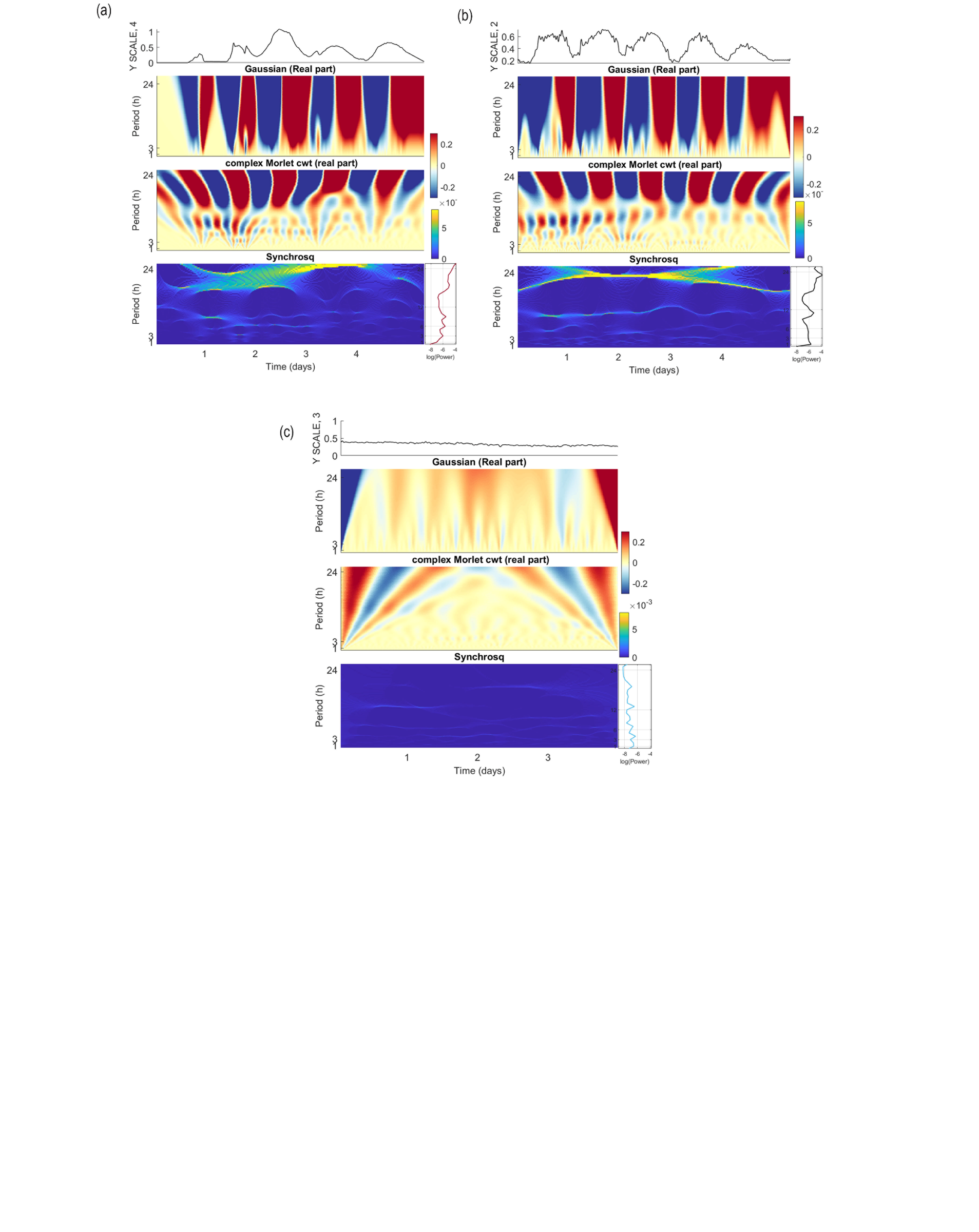


Supplementary Figure 4. The GaMoSEC, method was applied of phototaxis in *Chlamydomas reinhardii* following growth in continuous light (LL, 13,000 lux) under photoautotrophic conditions using HSA medium. Phototaxis measurement was initiated at time 0 at which point light intensity was decreased to 3,000 lux. From left to right, cultures were in early-, mid-, or exponential or stationary phase of growth; temperature, 28^o^C throughout. The time series are the same as shown in Fig. 4d-f, and Figure 6. For each time series the first three steps are shown, namely the Gaussian continuous wavelet transform, cwt, the real part of the complex Morlet cwt and Synchrosqueezing. In this last analysis, power was estimated for each hour window from the Syncrosqueeze scalogram (see comparison between conditions in Supplementary Figure 4) and shown in the left panel inset. Yellow horizontal bands in panels “b” indicate the presence of ~24h rhythms. In “a” this ~24h is not localized in frequency (period is not constant over time) and is not observed at all in panel “c”.


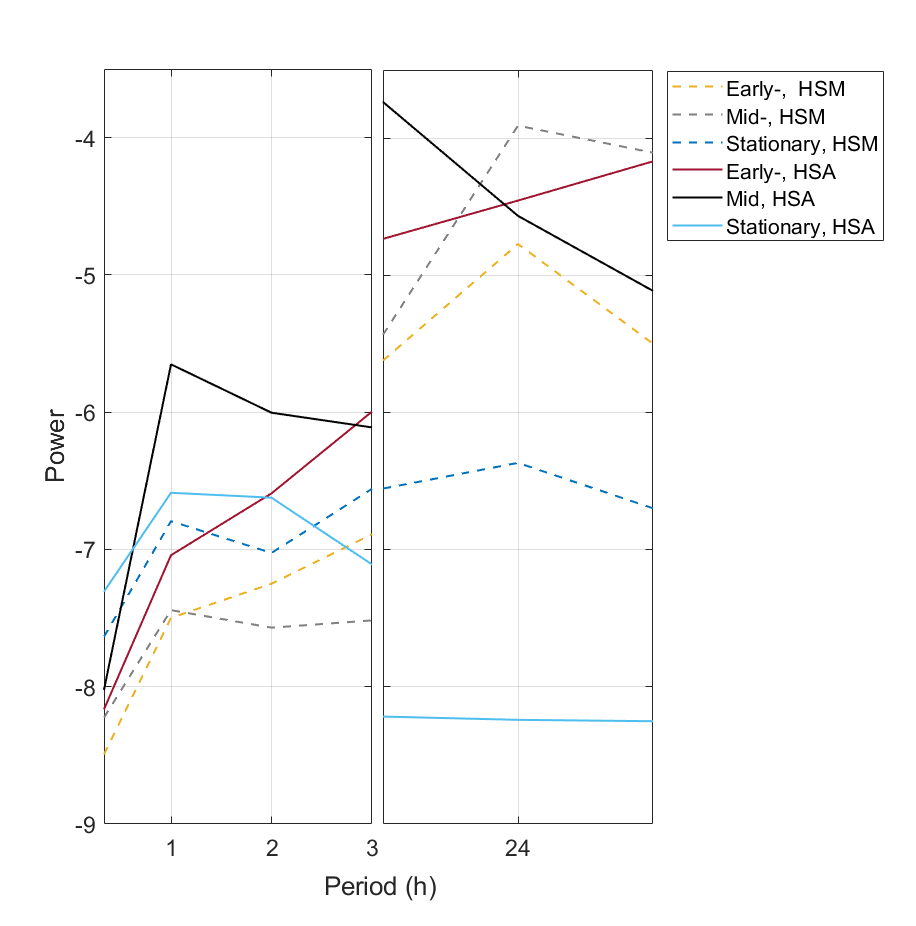


Supplementary Figure 5. Comparison of power of fluctuations between conditions, medium (i.e. HSM, H SA) and phase growth (i.e. early-, mid-, or exponential or stationary) of phototaxis in *Chlamydomas reinhardii.* The mean value of the maximum values of power was estimates for each hour window from the Syncrosqueeze scalogram. These represent the same analysis shown in Supplementary Figures 2 and 3. For simplicity two scaling regions are shown, consistent with visual observation of the time series, mid- phase growth under HSA conditions (black line) shows that highest level of fluctuations around the 1-2h scale, almost an order of magnitude higher than the other groups in the same medium, and almost 2 orders of magnitude higher than the early- and mid- phase growth under HSM medium. In regard to the circadian 24h scale, rhythms are strongest in the early- and mid- growth phase (black and red lines). Note that rhythms are not highly localized in frequency.
